# Medical education system (re)building in a fragile setting: Northwest Syria as a case study

**DOI:** 10.1371/journal.pgph.0001340

**Published:** 2023-04-11

**Authors:** Yamama Bdaiwi, Safwan Alchalati, Ammar Sabouni, Munzer Al-Khalil, Omer Abdrabbuh, Ahmad Kejah, Abdulhadi Shaban, Mohammad Almousa, Hasan Almarei, Abdalhakem Haj Asaad, Abdulhaseb Alkdro, Mohammed Almoayyad, Omar Waled Habboush, Preeti Patel, Abdulkarim Ekzayez

**Affiliations:** 1 Research for Health System Strengthening in Syria (R4HSSS) Project, King’s College London, London, United Kingdom; 2 Syria Development Centre (SDC), London, United Kingdom; 3 Syrian Board of Medical Specialties (SBOMS), Idlib, Syria; 4 Research for Health System Strengthening in Syria (R4HSSS) Project, Union for Medical and Relief Organisations (UOSSM), Gaziantep, Turkey; 5 Cerrahpasa Faculty of Medicine, Istanbul University-Cerrahpasa, Istanbul, Turkey; 6 Aleppo University, Aleppo, Syria; 7 Damascus University, Damascus, Syria; 8 Idlib University, Idlib, Syria; 9 Idlib Health Directorate, Idlib, Syria; 10 Syria Public Health Network, London, United Kingdom; Royal Infirmary of Edinburgh and University of Edinburgh, UNITED KINGDOM

## Abstract

**Background:**

Syria has witnessed more than a decade of armed conflict through which healthcare workers and facilities have not only been affected, but targeted. Amidst this targeting of healthcare workers, subsequent displacement, and ‘weaponization’ of healthcare, the medical education and health professional training (MEHPT) of those that remain has split into at least two distinctive contexts: government controlled, and non-government controlled. Efforts to rebuild MEHPT in light of this polarisation and fragmentation have led to a new MEHPT system in non-government controlled northwest Syria, that functions through what we describe as a ‘hybrid kinetic model’. This mixed-methods study provides an in-depth analysis of this MEHPT system as a case study to inform future policy planning and interventions in the context of future post-conflict health workforce development.

**Methods:**

We used mixed methods to investigate the state of MEHPT in northwest Syria during September 2021 and May 2022. This included a) Stakeholder analysis, b) 15 preparatory experts consultations c) 8 Focus group discussions d) 13 Semi-structured interviews e) 2 Questionnaires and f) Validation workshops.

**Results:**

We identified three main categories of key stakeholders working on MEHPT in northwest Syria: 12 newly established academic institutions, 7 local governance authorities involved in MEHPT, and 12 non-governmental organisations (NGOs). The MEHPT system operated through these stakeholders in a three-layer system to provide undergraduate and postgraduate MEHPT. In the first, top, layer, external NGOs and donors hold the strongest capacity at the expense of relatively under resourced internal governance in the second, middle, level. In the third, bottom, level, local academic bodies operate. We uncovered several levels of challenges facing these stakeholders including governance challenges, institutional challenges, individual challenges, and political challenges. Despite these challenges, participants in our study highlighted significant opportunities within the MEHPT system and that MEHPT can be a peace building pillar for the community.

**Discussion:**

To our knowledge, this is the first paper that provides an in-depth situational analysis of the MEHPT system in a conflict setting while engaging the voice of local key stakeholders. We found that local actors in MEHPT in non-government controlled northwest Syria have made efforts towards (re)building a new, hybrid and kinetic MEHPT system, through a bottom-up approach. Despite these efforts, the MEHPT system remains fragile and polarised, suffering from several levels of challenges with limited involvement of internal governance. Building on our findings, to improve this approach and build bridges of trust among stakeholders and the MEHPT community, further studies are needed to determine feasible approaches to increasing the role of internal governance structures in the MEHPT system through: 1-Formalisation of efforts through establishing a MEPHT technical coordination unit. 2-Further shifting of power from external supporting NGOs and funders to internal governance structures. 3- Working towards achieving sustainable long-term partnerships.

## Introduction

The decade long armed conflict in Syria has devastated all aspects of life in the country, including healthcare [1] Healthcare workers (HCWs) have been “weaponised” and purposefully attacked as a strategy to deprive civilians of essential services [1,2]. Since March 2011 and as of February 2022, Physicians for Human Rights (PHR) has documented 601 attacks on 44 separate healthcare facilities in Syria and the killing of 942 HCWs, most of which (90%) are thought to be committed by the Syrian government and their allies [2]. It is estimated that as many as half of the pre-conflict physicians in Syria, which was reported at 29,055 in 2007 by the Damascus Ministry of Health [3], have been displaced and by 2013; and up to 70% of the healthcare workforce had left the country, with hundreds more incarcerated or tortured [2,4]. In 2019, the United Nations estimated that half of the healthcare facilities in Syria were either destroyed or only partially functional [5].

As a result of the complex geopolitical situation in the country, there are two distinct territories in Syria that correspond to two main areas of political control. Around 60% of Syrian territories, including the capital Damascus and most central and southern areas, are controlled by the Assad Regime or government controlled (GC). GC areas are home to a population of around 10 million people. Non-government controlled (NGC) areas constitute the remainder of the country and house a further 8 million in the northwest and northeast of the country. The northeast NGC areas are under the control of the Kurdish-majority Self Administration of North East Syria (SANES), supported mainly by the United States, and have a population of approximately 4 million. The northwest NGC areas are controlled by various opposition armed groups, supported mainly by Turkey, with a population of a further 4 million [6].

The medical education and health professional training (MEHPT) sectors have echoed this geopolitical divide into at least two distinctive contexts; government controlled (GC) and non-government controlled (NGC) [7]. Each context has its own characteristics in terms of healthcare services and MEHPT realities [7]. Notably, the health system in NGC areas, particularly northwest Syria, functions through a hybrid semi-autonomous local health authority model which is very distinct to the centralised health system in GC areas [6]. In 2019, the University of Cambridge published a report with the non-governmental organisation (NGO) CARA- Council for At-Risk Academics–on the state of higher education in Syria. The report on Education in Syria documents the insecurity and risk that academics and students are currently confronted with given the heightened and intense politicisation. The report goes on to define politicisation as the "varying forms of political activity and ideological commitments relative to HE, depending on whether areas are under regime control or not [8]."

Despite the total disruption of MEHPT in NGC Syria and NW Syria caused by the conflict, this deficiency has been partially assuaged through the implementation of short-term medical training programs offered predominantly by humanitarian organisations and newly established local medical schools and health institutes. [9,10]. Tele-education and training have provided another substitute for traditional medical education for students and trainees whose education was interrupted due to the war, siege, or unsafe travel [11]. For example, NGOs have structured educational webinars in key medical fields for trainees and medical practitioners under siege [4], and surgeons have recorded procedures on their smartphones and posted videos on the internet so that they can be used in training [4,12,13].

Globally, since the cessation of World War II in 1945, there have been over 300 episodes of armed conflict in 151 locations across the world [14]. Nonetheless, there is a dearth of knowledge regarding the effect of contemporary conflicts on medical education in emergent health systems. Academic research regarding the implications of medical education and training in active protracted conflict contexts is notably scant. For example, after more than 20 years of conflict in the DRC, the medical education and training sector is still underdeveloped with major implications on quality of care and little understanding of the gaps and the way forward [15]. In comparison, there is more attention for medical education in post-conflict contexts. In Bosnia, shortly after the conclusion of the conflict, there was an increased focus on the necessity of reforming the medical education system in the country [16], with subsequent studies conducted to evaluate this sector and suggest further measures [17]. It has been argued that medical education and investments in this sector had a positive effect on peacebuilding in the country [18]. Drawing lessons from post-conflict contexts is of paramount importance in order to pay more heed to the examination of MEHPT adaptation mechanisms and to probe effective strategies of health workforce reinforcement amidst ongoing conflicts, in order to bridge the humanitarian-peace-development continuum [19].

In this study, we aim to provide a detailed in-depth analysis of the current situation of the MEHPT sector in northwest Syria as an example to inform policy planning and interventions in the context of future post conflict health systems strengthening.

## Methods

### Approach

We followed a mixed-methods approach as part of the Research for Health System Strengthening (R4HSSS) project [6] in north-west Syria at King’s College London and the Union of Medical Care and Relief Organisations (UOSSM), which included; a) a stakeholder analysis, b) Experts consultations c) Focus group discussions d) Semi-structured interviews e) Questionnaires and f) Validation workshops.

### Stakeholder analysis

Given the complex setting and the multiple key actors, we planned to identify and engage with key stakeholders. We followed a snowballing approach to identify and map the list of key stakeholders according to their role, type, and level of engagement in MEHPT sector.

### Experts’ consultations

We conducted 15 preparatory consultation meetings between July and August 2021 with representatives from the identified stakeholders to further understand the context and optimise the design of the interview guide and questionnaires.

### Semi-structured interviews

We then finalised the design of the interview guide and the interviewees list. The interviews were conducted in the Arabic language as the main spoken language in Syria. 13 semi-structured interviews were conducted between September and October 2021 via Zoom online platform, each interview lasted 40–60 minutes. The interviews were not recorded due to security concerns expressed by the interviewees.

### Focus group discussions (FGD)

8 FGDs were carried out and recorded online via Zoom platform with the help of a facilitator and 3 researchers (YB, MK, and SA) in September and October 2021. Each FGD lasted for 45 minutes, and included 4–7 representatives of the stakeholders. The FGDs explored the challenges, opportunities, learnt lessons from previous experience, future recommendations for policy planning and interventions.

### Questionnaires

Two questionnaires were sent between September and October 2021. The first questionnaire targeted the deans and heads of the academic bodies to collect data about the academic bodies’ curricula, staff, students, trainees, etc. And the second questionnaire was sent to the programme directors of the NGOs and governance bodies. In total 19 responses were collected electronically or in person with the help of our in-country data collection team.

### Analysis

A Thematic analysis approach was followed to analyse the qualitative data. Notes from recorded and unrecorded interviews were analysed. Codes were extracted by two bilingual (Arabic and English) researchers (YB) and (SA), and themes were developed through several meetings and discussion between the two researchers. Questionnaires’ data were grouped, categorised and narratively presented to best serve the aim of the research question.

### Ethics

Ethical approval was gained from King’s College London, and locally an approval to conduct the research activities was gained from both Idlib Health Directorate and Aleppo Health Directorate. Research information sheets, and consent forms were approved verbally for the FGDs and in-writing for the interviews. Further validation workshops took place online via zoom in April and May 2022 including representatives of all research participants. Additional information regarding the ethical, cultural, and scientific considerations specific to inclusivity in global research is included in the Supporting Information.

## Results

**Fig 1:**
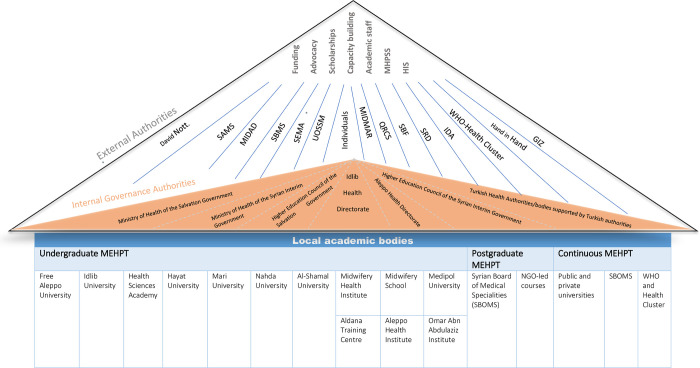
Key actors filling the void in health education governance.

We identified four key categories of MEHPT stakeholders in northwest Syria **(see Fig 1 and Table 1)**:

1. Higher academic institutions (12: 8 public and 4 private).
2. Health governance bodies (9).
3. NGOs (14).
4. Prominent individuals who are active in MEHPT leadership in northwest Syria.

**Table 1:** Stakeholders involved in medical education and health professional training (MEHPT) in northwest Syria.

**Governance bodies**													
**Aleppo areas**										**Idlib areas**			
Aleppo Health Directorate	Higher Education Council- Syrian Interim Government	Aleppo Ministry of Health—Syrian Interim Government	Ifrin health Directorate	Al-Bab Health Directorate	Jarablus Health Directorate	Al-Rai Health Directorate	Izaz Health Directorate	Idlib Health Directorate	Ministry of Health–Salvation Government	Higher Education Council—Salvation Government			
**Academic bodies**													
**Undergraduate MEHPT**										**Postgraduate MEHPT**			**Continuous MEHPT**
Free Aleppo University	Idlib University	Health Sciences Academy	Al-Hayat University	Mary University	Al-Shamal University	Midwifery Health Institute	Midwifery School	The Syrian Board of Medical Specialities (SBOMS)	Courses led by NGOs	Courses led by SBOMS, universities and institutes, NGOs, and WHO Health Cluster		
			Medipol University	Omar Abn Abdulaziz Institute	Nahda University	Aldana Training Centre	Aleppo Health Institute					
**NGOs and Individuals**													
Syrian American Medical Society (**SAMS**)	Syrian Expatriates Medical Association (**SEMA**)	Union of Medical Care and Relief Organisations (**UOSSM**)	Syrian British Medical Society (**SBMS**)	German Society for International Cooperation (**GIZ**)	MIDMAR	David Nott Foundation	Hand-in-Hand for Syria	WHO Health Cluster	Syria Bright Futures (**SBF**)	MIDAD	Qatar Red Crescent (**QRCS**)	Syria Relief and Development (**SRD**)	Independent Doctors Association (**IDA**)

As seen in **Fig 1**, the MEHPT system in northwest Syria is a three-tier system. The first tier represents local higher academic institutions, where MEHPT activities are conducted. The second tier is that of health governance. This is where the responsibility of local accreditation and a certain degree of oversight and coordination lays. Third is the local and international NGO level, where funding, capacity strengthening, scholarships, health information system support and advocacy activities are planned and achieved.

The relationship between stakeholders is complex. This complexity reflects how the MEHPT system is currently operating. This is more apparent in terms of the functions of local accreditation, funding, and governance. As an example of the degree of complexity at the level of local accreditation; higher academic institutions are forced to Acquire local accreditation from several health governance bodies, for the same function: the Free Aleppo University, for example, is accredited by the Higher Education Council of the Syrian Interim Government which is the Turkey-backed governance structure in areas in northern Aleppo under Turkish control (Olive Branch and Euphrates Shield areas) and allows these graduates to work in hospitals that are supported by non-governmental organisations in this area. This does not allow graduates however to practice within hospitals that are directly supported by Turkish authorities, as only medical personnel with accreditation from Turkey can practice within them. Additionally, for medical graduates from Free Aleppo University to practice in areas under Hayet Tahrir Alsham (HTS) in Idlib, they must pass a national exam from the Ministry of Higher Education of the HTS-affiliated Syrian Salvation Government.

The Syrian Board of Medical Specialities (SBOMS) also receives its main accreditation from the Syrian Interim Government (Ministry of Health) but is also affiliated with the ‘independent’ central desk that is the Idlib Health Directorate. Additionally, SBOMS may be forced, at some indirect and unofficial level, to coordinate with the Syrian Salvation Government. Furthermore, some health and midwifery institutes in Idlib and Aleppo obtain their accreditation directly from the Idlib Health Directorate.

Finally, Idlib University and some private universities in areas under HTS obtain their accreditation from the Syrian Salvation Government (Ministry of Higher Education); as does the newly formed Syrian Higher Council for Medical Specializations which obtains its accreditation from the Ministry of Health of the Syrian Salvation Government **(see Table 1).**

### Challenges and opportunities

The focus of this section relates to how MEHPT actors in northwest Syria identified the challenges they face providing MEHPT activities and the potential opportunities to overcome these challenges.

**Fig 2:**
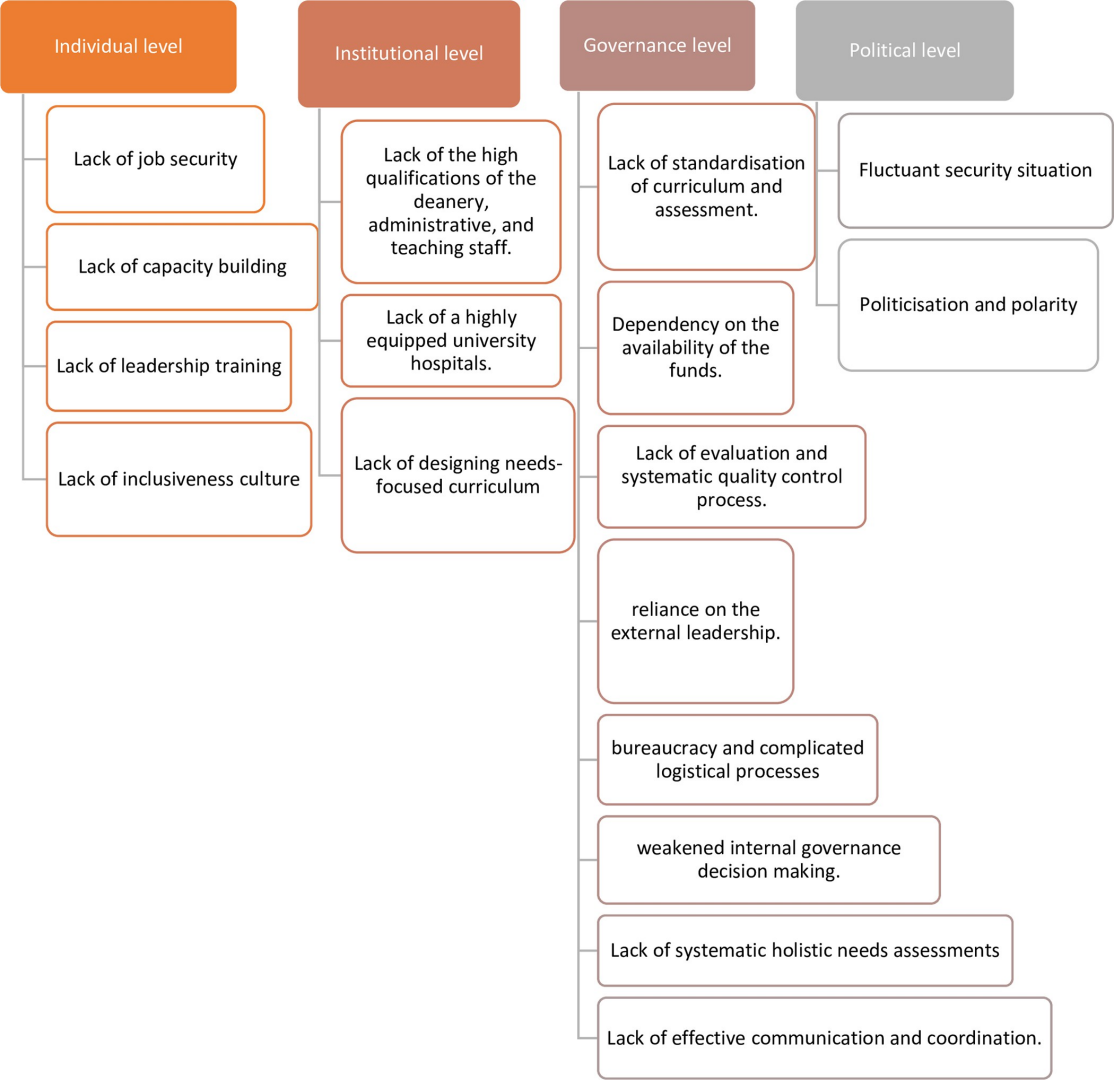
Challenges facing the MEHPT system.

The thematic analysis of the focus group discussions (FGDs) elicited key concepts relating to challenges at four levels. As may be expected, participants demonstrated overlapping understandings of these four levels of challenges: individual, institutional, political, and governance level. This overlap suggests a good interpretation of understandings and attitudes that is based on relative rather than isolated concepts. **(See Fig 2).**

#### Challenges

*Individual level*. Participants said they suffered from a lack of job security due to institutions relying on ‘on-and-off’ funding. This was compounded with a lack of continuous professional training in key areas they felt they needed including leadership and specialised training courses. Furthermore, they expressed significant distress from the lack of transparency and inclusivity in the work culture within MEHPT. Participants said this was due to ‘polarisation’ and even ‘politicisation’ within the work of institutions in the field.

“Our trainees suffer from the lack of trust from one side due to the almost absence of trainers who hold MSc and PhD certificates. They also suffer from feelings of injustice from the other side. When they struggle to get job opportunities due to the quantity–labour mismatch and lack of curriculum and assessment standardisation among the different academic bodies.”

             *Director of an academic body*

*Institutional level*. Participants expressed facing challenges at the institutional level due to the lack of capacity strengthening and specialised qualifications of teaching staff, and even senior management and leadership. Additionally, participants described difficulties tailoring need-focused curricula for their institutions given the lack of local standards and policies.

"We rely on the lecturer’s skills and knowledge rather than set curricula. Some of our modules are taught based on translated textbooks that are 30 and 35 years old which are far from being related to the health needs of our people or even the research community.”

             *Senior faculty member of an academic institute*

“We are not used to conducting research, we have a lot to tell, but we are not even trained on how to write a scientific paper. We need interventions for our cadres rather than on our facilities and infrastructure.”

             *Representative of a governance body*

*Political level*. The unstable state of security, ‘polarisation’, and ‘politicisation’ of MEHPT were the major factors impacting support channels and reaching out to donors. Participants felt these issues were a real impediment to achieving a stable base for development work.

“It is time now to neutralise medical education work, as we were able to neutralise medical relief and humanitarian work when the situation and the context necessitated it, now it is time to do this for medical education because the situation and the context necessitates.”

             *Representative of a national NGO*

*Governance level*. Participants expressed major challenges facing them on the governance level due to the weak internal decision-making capacity of local governance bodies. The reliance on external funding channels from donors and non-governmental organisations (NGOs) has caused a lack of autonomy and independence of these bodies. Furthermore, this has been the cause for complexities related to the requirements of donor-related reporting, red tape, and logistics. Participants also described this as the reason for a ‘hesitance to initiate change’:

“There is no unified governance structure that can coordinate efforts, set plans, achieve standardisation, achieve effective external partnerships, and approach funders. This creates negative competitiveness between the local bodies for funding opportunities which come and go, and projects are hugely dependent on the presence of external funding, as long as there is a donor organisation implementing the project and providing funds, the project will continue, as soon as it stops, the work ends. This is a waste of local efforts, and a source of disappointment.”

             *Representative of a governance body*

Given the subsequent lack of holistic and systematic need assessment studies to inform the initiation and distribution of support programmes, there is a lack of effective coordination and communication towards standardisation of curricula, assessments, evaluations, and quality control procedures.

“Lack of local accreditation is a significant issue in northwest Syria, there is a focus on international recognition and external accreditation, at the expense of the local accreditation—I will only accredit your graduates if you accredit my graduates.”

             *Director of academic institute*

#### Opportunities

Despite the multi-level challenges facing actors, participants perceived significant opportunities within the MEHPT sector. They said they believed the MEHPT has high levels of resilience. They also expressed a belief in a dominant culture of proactivity and creating positive changes which they felt would be the catalyst for future development work. They also professed a strong sense of loyalty to MEHPT and mutual hope in bettering the future of health and creating peace for the local population. These feelings stemmed from their joint experiences during the ten plus years of “sacrifice”, “enduring extreme violence” and operating within limited or absent resource-settings:

“We work in a uniquely complex context, we have been able to establish institutions from scratch under shelling, what we need now is to look at medical education and training from a different lens, we need to go beyond short intermittent courses to the space of building systems and strengthening institutions. We need to re-think medical education and training as a peace building pillar for our community.”

             *Key individual*

The extensive efforts of the Syrian diaspora were seen as a significant pillar of support which “broadened horizons” for local actors. Additionally, innovation and technology made it possible to coordinate remote training and capacity strengthening activities for local staff by experts from international institutions. This was perceived to have created the potential to network and establish more stable and sustainable partnerships with international academic institutions.

The participants suggested several recommendations for future work in the field of MEHPT in northwest Syria. Firstly, they perceived the political neutralization of MEHPT as the main pillar for future development. They explained that this may include lining up around “a core body” or “a core alliance” which is able to overcome the polarisation and political sensitivities within the context and hence guarantee representation, independence, and collective decision making in MEHPT towards achieving sustainable and effective external partnerships. Secondly, strengthening the local MEHPT governance bodies to enable improved leadership, ownership, coordination, accreditation, and decision making. They suggested this may be achieved by gradually shifting power from external support systems (including NGOs and donors) to local governance systems, together with holistic capacity strengthening approaches and programmes in leadership, conflict resolution, management, strategic planning, information technology, needs assessment, public health planning, research and quality improvement, communication, “common value-building”, and English language skills. Thirdly, networking with neighbouring and international academic institutions and establishing sustainable academic partnerships and capacity building programmes with local academic bodies, including training senior leadership and teaching staff especially in areas such as assessments, teaching strategies, curriculum design, and English language.

## In-depth MEHPT system analysis

### Local governance

**Fig 3:**
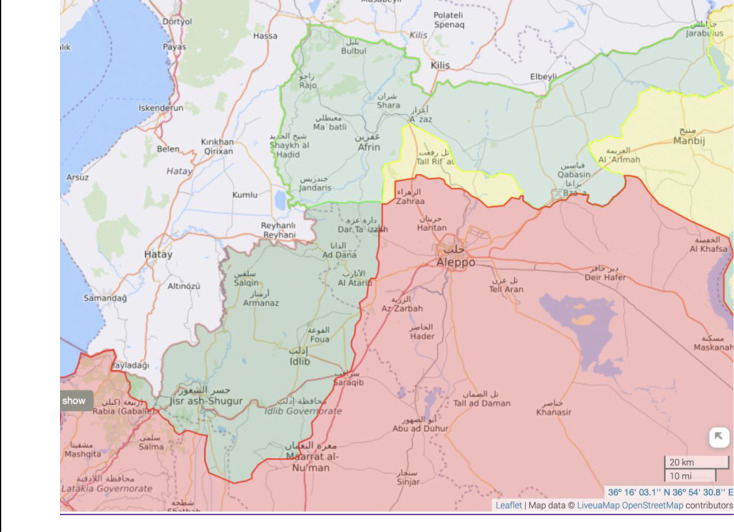
Northwest Syria areas of control: Areas under Turkish control are in light green, areas in darker green are under the control of Hayat Tahrir Sham (HTS). The full map can be accessed at https://syria.liveuamap.com. Copyright https://syria.liveuamap.com/about#terms.

Northwest Syria comprises two distinct areas of control: areas under Turkish control (opposition groups backed by Turkey–otherwise referred to as Euphrates Shield and Olive Branch areas) in northern Aleppo (light green in Fig 3) and areas under the al-Qaeda affiliated Hayat Tahrir Sham (HTS) in Idlib (darker green in Fig 3). These two areas correspond to two governments: Syrian Interim Government, and Syrian Salvation Government, respectively.

Within the areas of control of the Syrian Interim Government (SIG), there exists the Ministry of Health of the SIG, and the Council of Higher Education of the SIG, in addition to several health directorates which are supported by the Turkish Health Authorities: the Ifrin Health Directorate, Al-Bab Health Directorate and Izaz Health Directorate **(see Table 2)**. The Council of Higher Education of the SIG supervises the recent programme for postgraduate medical training within the Free Aleppo University and the Turkish Health Authorities oversee some limited MEHPT activities in areas of northern Aleppo including Ifrin, Al Bab, Jarablus, Al Rai, and Izaz. Within the areas of control of the Syrian Salvation Government (SSG), there exists the Ministry of Health of the SSG, and the Ministry of Higher Education of the SSG. The Ministry of Health of the SSG supervises the newly formed Syrian Higher Council for Medical Specializations which operates within Idlib University.

**Table 2:** Local governance bodies supporting medical education and health professional training (MEHPT) in northwest Syria.

Local governance body	Aleppo Health Directorate	Idlib Health Directorate
**Year of establishment**	2013	2013
Historical role prior to the start of the Syrian conflict	N/A	N/A
**Current role**	Supervises and runs MEHPT activities and provides a governance umbrella of the available local expertise and holds the responsibility of their qualifications, training and needs assessment.	Supervises several training programs and speciality professional diplomas- Prepare health system action plans-
**Area of expertise **	All medical staff including undergraduate and postgraduate and CME.	Hospitals, primary health care centres, specialised centres- CME
**Current interventions**	Training programs for the HCWs- Currently working on establishing an affiliated institute.	Supervises several health institutes to qualify HCWs- runs annual training and assessment programs
**Aims of the current interventions**	To improve the experience of HCWs and improve their qualifications to up-to-date standards.	To improve the experience of the HCWs and provide them with up-to-date guidelines.
**Main challenges**	The absence of financial support- Lack of co-operation with the NGOs.	Absence of annual financial stability to achieve sustainable training programs- Challenges in running recruitment assessments at the health facilities due to their affiliations with different NGOs with different local policies.
**Coping strategies**	Working towards gaining a sustainable financial stability and support- Aiming to coordinate and better the coordination with all the active NGOs to agree on action and strategic plans.	Working towards gaining alternative financial support channels- Reaching out and networking with health facilities.
**Recommendations**	Advocating to establishing a university hospital- Strengthening the role of the health directorates- Regular meetings with all active stakeholders.	Continue to run training courses with the coordination with the NGOs
**Types of support needed **	Financial support- External partnerships to build capacities	Stable financial support- Networking with external academic bodies and governance bodies to help building capacities.

In addition to these ‘formal’ structures, two further locally led governance structures exist with a slightly larger margin of independence from the government structures and are able to operate across areas of control: Aleppo Health Directorate and Idlib Health Directorate. Aleppo Health Directorate (AHD) was established in 2013. It is technically part of the SIG, and oversees MEHPT activities for undergraduate, postgraduate, and continuous medical education of healthcare workers in the northern Aleppo countryside areas (including Ifrin, Euphrates Shield, Al-Atareb, and Daret-Azza).

Idlib Health Directorate (IHD) was established in 2013. In terms of MEHPT, IHD aims to enhance and support the qualifications and standards of practice of healthcare workers and oversees training programs and speciality professional diplomas in the Idlib Governorate (including Idlib, Ariha, Harim and Jisr Ash-Shugur). Although it is a technically a part of the SIG, it maintains a large margin of independence due to its significant presence in SSG areas. IHD covers primary health centres, secondary centres, and specialised hospitals in the area.

The main challenges facing both local health directorates (Idlib and Aleppo) as expressed are a) the absence of annual financial stability to achieve sustainable training programs, b) the lack of effective strategic and implementation co-ordination with the health facilities due to affiliations with multiple NGOs and different policies, and c) the lack of capacity, leadership and managerial training of the Directorate staff.

### Non-governmental actors

In our analysis, we were able to identify 14 non-governmental organisations (NGOs) supporting the MEHPT sector in northwest Syria including; the SAMS [20], SEMA [21], UOSSM [22,23], SBMS [24], QRC [25], SBF, SRD [26], David Nott Foundation [27], Midmar [28], Hand-in-Hand for Aid and Development (HIHFAD) [29], IDA [30], GIZ [31], Midad [32] and the WHO Health Cluster and the WHO Health Cluster (we did not collect data from the latter 3 organisations due to logistical issues). **(See Table 3, and for further details: S1 Appendix).** Most of the Syrian NGOs were established after the start of the Syrian conflict, the remainder had a very limited role in the MEHPT sector prior to the Syrian conflict. The aims of theses NGOs in the MEHPT sector are mainly to developing the health system in northwest Syria, through a) providing financial support to the MEHPT academic institutions through international advocacy, b) delivering capacity strengthening support to the medical community and cadres through scholarships, short courses, and direct advice, and c) supporting MEHPT academic institutions with experienced teaching and training staff, utilising hybrid and online platforms. The main challenges that were believed to be detrimental to the work of these NGOs in the MEHPT sector are a) the lack of international accreditation of certifications, b) the limited role of local governance bodies, c) difficulties in securing and sustaining funding from donors for MEHPT sector, d) the unstable security situation, and e) logistical complexities due to the lack of organisation and co-ordination amongst actors. **(See Table 3, and for further details, S1 Appendix).**

**Table 3:** Non-governmental organisations (NGOs) supporting the medical education and health professional training (MEHPT) sector in northwest Syria.

NGO	Role and beneficiaries in relation to MEHPT system	
	Educational and financial support	Capacity strengthening and scholarship support
**Syrian American Medical Society (SAMS)**	Undergraduate Midwifery and Nursing Health Technical Institute in Al-Atareb. The Syrian Board of Medical Specialities (SBOMS). Psychological support workers and psychotherapists (Vocational training). (Upcoming) psychologist, psychiatric nursing, and psychosocial support worker training programme.	Health sciences student scholarship program in neighbouring countries.
**Qatar Red Crescent (QRCS)**	Academy of Health Sciences in Qah. (Previously) medical residency program of SBOMS through a partnership with Syrian Expatriates Medical Association (SEMA).	20 MSc. scholarships at Ankara Yildirim Bayezid University in Turkey in public health and health policies in collaboration with SEMA. Training health directorate and organization staff in epidemiology.
**Syria Bright Future (SBF)**	Mental health and research projects. Advanced problem-solving programme, as per the third level of the pyramid of psychosocial interventions.	Strengthening managerial and leadership capacities and life skills. Training on protecting and empowering women, children, and youth.
**Syria Relief and Development Foundation (SRD)**	Midwifery school. Neonatal and Childhood Illness training.	Capacity strengthening for foundation staff in different health topics based on WHO health programs.
**David Nott Foundation**	Surgical courses with postgraduate education for surgeons, educational online group, in addition to medical advice, and surgical expertise.	-
**Union of Medical Care and Relief Organisations (UOSSM)**	(Upcoming) psychiatry training programme with SBOMS.	Training staff on programmes and protocols in global health.
**Hand-in-Hand for Aid and Development (HIHFAD)**	(Previously) supporting a residency program in coordination with SBOMS. In 2017, supported a project to qualify 120 student nurse assistants.	-
**Independent Doctors Association (IDA)**	Supporting a training centre in BAS—Border side.	-
**The Syrian British Medical Society (SBMS)**	Expert advice through UK diaspora consultants. Advanced surgical and trauma courses in coordination with Royal Colleges.	-
**Syrian Expatriates Medical Association (SEMA)**	Vocational health education through: • The Academy of Health Sciences • SEMA Vocational Health Education Centre • Safe Neonatal Emergency Transport Program (SENT Program). • Medical Education Vocational Diploma Program • Cooperation with the Turkish Red Crescent to establish a new vocational health education institution within Syria. Continuing medical education (CME) through paediatric residency lecture series.	Financial support for 400 undergraduate students to graduate, in addition to career advice and job fairs. 20 MSc. scholarships at Ankara Yildirim Bayezid University in Turkey in public health and health policies in collaboration with Qatari Red Crescent.
MIDMAR^1^	Previous experience within UOSSM that extended from 2013 to mid-2017, Providing a range of specialized services in CME including training programs, quality diplomas and response projects. Currently supporting a project to qualify 125 specialised nurses (intensive care—incubator care—surgeon assistant–dialysis)^2^ Implementation of service contracts for medical software for medical institutions operating in northwest Syria.	-

^1^ https://midmar.org/developing-activities-training/.

^2^ https://www.uossm.org/training.

### Postgraduate institutions

The main provider for postgraduate training is the Syrian Board of Medical Specialties (SBOMS) [33], which was established in 2015, and supervises the medical residency programs and grants a certificate called the “Specialization Certificate of Syrian Board of Medical Specialties” for trainee physicians in various specialties. The certification is granted after completion of examinations (including theoretical, practical and OSCE exams) and a training period at one of 35 health facilities approved by SBOMS, according to a set curriculum. Within SBOMS28 scientific committees with 175 members coordinate training (including teams of qualified specialists inside Syria and diaspora doctors). SBOMS is recognized by almost all health organizations and institutions in northwest Syria and doctors who have received the SBOMS certificate are considered specialists in Turkish hospitals located in Syria in the northern countryside of Aleppo. SBOMS aims to eventually obtain full accreditation by the Turkish Health and Education Authorities.

The scientific committees of SBOMS currently supervise 278 residents and have supervised 479 residents to date. SBOMS also carries out various continuous medical education (CME) activities, including scientific conferences, academic lectures, workshops, and courses through an online educational platform (https://cme.sboms.org/).

Postgraduate and CME training activities for other healthcare professionals are organised by various NGOs and undergraduate universities and institutes (such as Aldana Centre which provides nursing specialty training), in addition to the WHO and the health cluster **(see Table 4).**

**Table 4:** Syrian Board of Medical Specialities (SBOMS).

Postgraduate training body	The Syrian Board of Medical Specialties (SBOMS) [33]
**Year of establishment**	2015–2016
Historical role prior to the start of the Syrian conflict	N/A
**Current role**	“A scientific professional academic board that grants a unified certificate called Specialization Certificate of Syrian Board of Medical Specialties for all trainee doctors in various specialties after completing the training period curriculum which is accredited in the medical facilities approved by SBOMS and passing the required examinations.” SBOMS is an independent legal and financial identity. It is technically affiliated to the Ministry of Health of the Syrian Interim Government.
**Area of expertise **	Speciality training.
**Current interventions**	Currently includes 278 residents, historically 479 residents for speciality training through 28 scientific committees: Vascular Surgery, Urology, Paediatrics, Paediatric Surgery, Otorhinolaryngology, Orthopaedic Surgery, Ophthalmology, Oral and Maxillofacial Surgery, Obstetrics and Gynaecology, Neurology, Nephrology, Urology, Thoracic surgery, Neurosurgery, Psychiatry, Family medicine, Internal Medicine, General Surgery, Gastroenterology, Radiology, Dermatology, Adult intensive care, and Anaesthesia and intensive care. In addition to CME training.
**Aims of the current interventions**	• Set and supervise curricula and standards for training programs. • Set the standards for the recognition of medical facilities as training centres. • Conduct a needs assessment to identify shortages in medical specialties. • Set the standards for the supervising doctors and oversee them. • Launch selection criteria for admission to different specialties. • Grant the medical Specialization (called Specialization Certificate). • Conduct monitoring and evaluation of Resident doctors and their training. • Work on finding training scholarships abroad for Syrian students. • Contract agreements to obtain recognition of the certificate. • Organize workshops and conferences and plan scientific research.
**Main challenges**	Absence of international academic accreditation- Shortage of experienced senior speciality supervisors- limited clinical training venues such as highly equipped university hospitals.
**Coping strategies**	Networking to gain academic accreditation, diaspora senior speciality supervisors, networking to raise funds to improve the current training venues.
**Recommendations**	Academic partnerships to achieve academic accreditation- recruiting and training more senior speciality supervisors- partnerships with funders and NGOs- improving trainee experiences and wellbeing- achieving a neutral position from different stakeholders.
**Types of support needed **	Financial support for the speciality trainees and supervising staff- academic training and capacity building for the supervising and management staff- Establishing a well-equipped university hospital.

### Undergraduate institutions

According to our findings, there are 4 faculties of medicine, dentistry and pharmacy (2 public and 2 private faculties), 2 faculties of health sciences (1 public and 1 private), 5 nursing institutes, 4 midwifery institutes, 4 anaesthesia institutes, 2 physiotherapy institutes, 3 orthodontics institutes, 2 pharmacy institutes, 1 laboratory institute, 1 emergency institute and 1 paramedics institute. These are located between non-government-controlled areas of Aleppo and Idlib, and are subordinate to two governmental bodies (SIG and SSG). These faculties and institutes provide undergraduate teaching to more than 6365 students. The funding sources vary between direct donations, NGO funding, and students’ registration fees, although most participants refused to reveal detailed financial information. The registration fees vary between free or 50$ per year up to 3000$ per student per year. The number of deanery staff per institution varied between 2–10 people, some of whom are qualified at MSc-PhD level, but with very limited training available. The number of managerial staff varied between 8–17 per institution, with limited training on management. The number of teaching staff varied between 22–50 per institution and regularly included diaspora expertise, with varying degrees of qualification from bachelors, diploma, MSc, and PhD, with very limited training on teaching strategies, methodology, assessment, scientific research methodology and medical education.

The teaching approaches implemented within undergraduate institutions are seminars, laboratory sessions, lectures, interactive sessions, simulation and clinical training, and the curriculum used is either based on the translation of textbooks, public Syrian University curricula from before the conflict, or coordinated individually by teaching staff. It was not uncommon for some modules to be cancelled due to lack of teaching staff, particularly scientific research modules. The enrolment process is based mainly on the high school graduation results, and assessment is based on both theoretical and practical exams. The infrastructure includes venues equipped with lecturing theatres, seminar rooms, labs, online platforms, and contracts with hospitals to run clinical training. Most of the undergraduate universities and institutes were established around the year 2015 and have been operating continuously since then **(see Table 5, and for further details: S1 Appendix).** In addition to the insitutitons in the tables, more recently, some Turkish universities have launched branches in northwest Syria, such as The University of Health Sciences, Medipol University and Gaziantep University, in addition to some health institutes such as Aleppo Health Institute, Aleppo Technical Institute, and the Midwifery School.

**Table 5:** Undergraduate MEHPT universities.

Academic body	Type	Faculties/institutes	Number of students	Infrastructure and equipment
**Free Aleppo University [34]**	Undergraduate, public	Faculty of medicine, faculty of dentistry, faculty of pharmacy, faculty of health sciences, anaesthesia, nursing, and midwifery institutes.	More than 2100 students	Lecturing theatres, seminar rooms, labs, hospital-based clinical training sites.
**Nahda Private University [35]**	Undergraduate, private	faculty of medicine, faculty of dentistry, faculty of pharmacy, faculty of health sciences. Pharmacy, radiology, orthodontics, paediatric nursing, biochemistry, and midwifery institutes	1000 students	Labs, hospital-based clinical training sites, online platform.
**Al-Hayat Private University**	Undergraduate, private	Faculty of nursing faculty of midwifery Aesthesia and physiotherapy institutes	Faculty of Nursing 242 Faculty of midwifery 111 Aesthesia institute 86 Physiotherapy institute 199	Seminar rooms with projectors, screens, whiteboards and mannikins.
**Health Sciences Academy [36]**	Undergraduate, public	Paramedics nursing, and physiotherapy institutes	Paramedics 40 Nursing 42 Physiotherapy 45	Lecture theatres equipped with projectors, screens, whiteboards and mannikins.
**Mary Private University [37]**	Undergraduate, private	Faculty of dentistry. Orthodontics, and dentist assistants’ institutes	500	Lecture theatres and labs
**Al-Shamal Private University [38]**	Undergraduate, private	Faculty of medicine, faculty of dentistry, faculty of pharmacy. Nursing, midwifery, emergency, and anaesthesia health institutes	2000 students	Lecture theatres, seminar rooms, labs, university hospital training.
**Idlib University [39]**	Undergraduate, public	Faculty of Medicine, faculty of pharmacy, orthodontics institute, pharmacy institute, and anaesthesiology institute	More than 1500 students	Lecture theatres, seminar rooms, labs, university hospital.

## Discussion

This study contributes to the expansion of knowledge regarding medical education interventions and their efficacy in conflict settings, particularly with regards to the politicisation of health and its effects on medical education. The case of Medical Education and Health Professional Training (MEHPT) initiatives in northwest Syria provides an example of a hybrid system that was developed using bottom-up approaches to invest in positive kinetic factors such as training interventions provided by humanitarian actors, active diaspora willing to support local initiatives, and the availability of highly educated health workers with high levels of social commitment to improve the health sector. These factors and this hybrid system for MEHPT can be further developed in the early recovery phase in northwest Syria, and may also serve as a resource for other conflict contexts from which to draw lessons.

Our findings include a stakeholder analysis of key actors working towards (re)building the MEHPT system in northwest Syria, and an analysis of the approach they have taken to (re)building. This work represents an analysis of the MEHPT system as it currently stands in northwest Syria, as of the end of 2021.

As of 2021, there have been significant geopolitical transformations in northwest Syria that have had a deleterious impact on the health sector and, consequently, the MEHPT hybrid system. The major challenges that began to emerge in 2020 and could shape the next phase of the humanitarian response in northwest Syria include: the dwindling territories controlled by opposition armed groups in northwest Syria, and thus the uncertainty regarding the future of this area in terms of political and military control. Secondly, the protracted nature of the conflict and the comparatively reduced hostilities on the one hand, and the emergence of other active conflicts, such as the one in Ukraine, all represented factors that diverted some of the humanitarian funding from northwest Syria. Additionally, the lack of clarity concerning the governance of northwest Syria, compared to the more stable governance in the other areas of control, meant that some donors started to shift their attention away from northwest Syria. All of this constituted novel challenges that the MEHPT must adapt to in order to cope with the new difficulties. Nevertheless, we believe that the lessons learnt, and the cumulative experience will inform the next steps of MEHPT in the region and possibly in the other areas of control in Syria.

### Actors (re)building MEHPT in northwest Syria

The protracted armed conflict in northwest Syria burdened the previously fragile medical education and health professional training system to the point of breaking, with weaponization of healthcare, politicisation, and loss of physical and human resources. The following actors have played a role in the (re)building of the MEHPT system:

1. Fourteen national and international NGOs providing external support to:
2. Seven governance authorities which oversee the provision of MEHPT by:
3. Fourteen academic bodies covering undergraduate and postgraduate MEHPT in northwest Syria

In emergency phases of conflict settings, numerous external actors, including international agencies, donors, and non-governmental organizations (NGOs) concentrate efforts to provide assistance for collapsing MEHPT systems [40,41]. For example, 50 bilateral donors and multilateral agencies were actively involved in the West Bank and Gaza in 1999, and 60 donors and agencies were involved in Bosnia-Herzegovina during the same period [42]. However, following emergency phases, continued provision of health and MEHPT by NGOs and external actors can delay the development of indigenous health and governance systems [43]. These are potentially factors that the ‘early recovery’ or ‘development’ phase can inherit from the ‘humanitarian’ or ‘emergency’ phase of the conflict as our research has demonstrated.

### Approach to (re)building MEHPT in northwest Syria

The cumulative and collaborative work of these actors in medical education and health professional training (MEHPT) in northwest Syria has created a hybrid and kinetic MEHPT system through a bottom-up approach that mirrors how local health governance bodies were created in the region [44]. The system is hybrid in the sense that it is built around a complex network of relationships between intrastate (local institutions and experts), extra-state (foreign state authorities, aid agencies, diaspora experts, and diaspora non-governmental organisations), de facto (local health directorates) and de jure (ministries of health, and higher education) [45]. Non-governmental actors within this classification play a very substantial role including funding of institutions, design of training, human resource recruitment and training delivery. The system is kinetic in the sense that it is continuously changing, adapting to new programmes, funding, and realities. This (re)building of the MEHPT system is occurring within the vacuum left by the absence of alternative bodies to govern health workforce development in northwest Syria.

For this approach to continue to grow, stronger coordination mechanisms are needed. A successful example of effective and timely coordination mechanisms was seen in East Timor, as early implementation of a sector-wide approach [46] was the foundation stone for guaranteeing appropriate coordination and enhancing legitimacy. It placed Timorese at the centre of the process, with the external actors supporting them with technical and financial assistance. Such a transition, if achieved in an equitable and sustainable approach is believed to contribute towards social peacebuilding in the local community, similarly to other post-conflict contexts [47]. In order to support a similar transition our participants have underscored the need to create a “neutral space” for actors to start coordinating with an increased level of ownership and support. Thereby “shifting the power” from extra-state supporting NGOs and funders to intra-state governance structures, working at the same time on strengthening their capacity through identifying clear mechanisms of coordination and implementation and also working on long term sustainable programmes rather than short term projects.

The effects of these efforts can build bridges of trust and respect. As war does not only destroy facilities, equipment, and human resources, but also trust, networks, and support, there is increasing evidence that improved provision of public services plays a significant role in the trust-building process, which then establishes conditions that lead to mutual confidence, respect, and reliability among actors [48,49]. This then increases the internal institutional readiness, and team cohesion, to accomplish goals in development [50]. Furthermore, within the sector of health workforce development in conflict settings, the coordination efforts are suggested to be formalised through the establishment of working groups of actors, and subgroups of units dedicated for certain scopes within health workforce development [51]. These units are suggested to be strongly embedded in the de-facto health authorities and governance structures, and lead on producing situational analysis, databases, policies, and finance strategies through establishing long-term sustainable external partnerships and the implementation of system-based designed approaches, with the support of the NGOs and diaspora health workforce [52].

This addresses the major challenge to this new system identified in our research which is “polarisation” (and sometimes “politicisation”). We define “polarisation” as a culture of limited coordination and negative segregation between actors based on their financial and sometimes political affiliations. According to our participants, factors that may have led to this polarisation include inequitable distribution of opportunities, lack of long term support programmes, heavy reliance on funding at the expense of weakened institutional decision making and a low internal governance coordination capacity. This is comparable to the situation in Uganda in 1986. The fragmentation of donors’ interventions including United Nations (UN) agencies, NGOs and bilateral donors was cited as responsible for hindering the development of a comprehensive policy for sustainable and equitable health development [53]. Therefore, the wide range of conflict setting experiences urges the necessity for rigid coordination mechanisms [54].

### Practical steps

All academic bodies in northwest Syria work solely in one area of geopolitical control. The only exception is the Syrian Board of Medical Specialities (SBOMS) which operates in areas controlled by both the Syrian Interim Government (SIG) and the Syrian Salvation Government (SSG). The rest of the academic bodies work solely in one of the two. It is therefore optimistic and unrealistic to advocate the formation of a single central body to coordinate MEHPT activities across areas of geopolitical control, when the political will does not exist for this to happen. There have been several unsuccessful attempts during the past years to form a unified higher education council. Unfortunately, all attempts collided with the “polarisation” and “politicisation” described above and failed. However, coordinating MEHPT activities at the levels of geopolitical control, in Idlib and Aleppo governorates separately, is perceived as possible as per our analysis. Therefore, to avoid duplication of efforts, wasting limited resources and to create a level of coordination to assure better quality and standards, we recommend the following policy steps:

1. Establishing a MEPHT technical coordination unit in Idlib and another in Aleppo, each to bring local governmental institutions, academic bodies, and independent actors together within formalised frameworks of action and financial strategies. This will help local initiatives in each area to learn from each other, coordinate interventions, avoid duplications, and gradually work towards joint planning exercises.
2. The establishment of a Working Group for MEPHT in Northwest Syria, which brings together leading local, regional, diasporic, and international actors involved in MEPHT activities, is essential for the development of effective strategies, the convergence of views among different stakeholders, research and evidence-based evaluation of MEPHT interventions, and the identification of gaps in support. In 2022, the R4HSSS Project initiated such a group with more than 22 members representing various local and international actors involved in MEPHT work. The Group is jointly coordinated by the R4HSSS Project, the Syria Development Centre (SDC), and the SBOMS, thus ensuring a balanced approach to navigating the complexities of the governance system in Northwest Syria. This Working Group could provide a pathway towards a transitional period for medical education, capitalizing on existing resources and initiatives, while providing evidence for mid- to long-term planning.
3. Agreeing on policies that guarantee shifting the support of NGOs from directly directing the academic bodies to supporting the technical coordination units to fulfil the emerging training gaps.
4. Working towards achieving long-term partnerships with academic and governance institutions regionally and internationally to strengthen the capacity of the units and their subordinate academic bodies.

### Limitations

During the conduction of this research, we faced numerous challenges, including difficulties in engaging with some actors due to the negative competition and political sensitivities in the context. Ineffective communication within the institutes increased the burden on our data collection team, for example in some instances the team had to visit some institutes multiple times because the institution staff were not informed about the team’s visit despite the meeting being scheduled by the institute representative beforehand. Lack of research participation culture was also a barrier due to the lack of awareness towards the potential positive impact of research on the medium and long-term. Transportation and long travel between the different sites in the country, logistical and technical difficulties, and the busy schedule of the participants were also a challenge to tackle. In addition to the inability to record the interviews due to security concerns expressed by the participants.

## Conclusion

We found that in a complex, conflict setting, rebuilding MEHPT system through a hybrid and kinetic bottom-up approach- although can fill a significant gap that is normally neglected- may remain fragile and polarised, unless strong involvement of the internal governance was achieved. Building on our findings, to improve this approach and build bridges of trust among stakeholders and the MEHPT community, further studies are needed to determine feasible approaches to increasing the role of internal governance structures in the MEHPT system through: 1-Formalisation of efforts through establishing a MEPHT technical coordination unit. 2-Further shifting of power from external supporting NGOs and funders to internal governance structures. 3- Working towards achieving sustainable long-term partnerships.

## Supporting information

S1 Appendix Detailed analysis of NGOs and academic institutions in northwest Syria. (DOCX)
