# Supplementary material for: Medical education system (re)building in a fragile setting: Northwest Syria as a case study
Source: PLOS Glob Public Health. 2023 Apr 11;3(4):e0001340. doi: 10.1371/journal.pgph.0001340 (PMC10089361; doi:10.1371/journal.pgph.0001340)
Supplement: S1 Appendix — (DOCX) [file pgph.0001340.s001.docx]

**S1 Appendix: detailed analysis of NGOs and academic institutions in northwest Syria:**

**Table A: Detailed analysis of the NGOs supporting the MEHPT sector in northwest Syria.**

| NGO | Syrian American Medical Society (SAMS) | Qatar Red Crescent (QRCS) | Syria Bright Future (SBF) | Syria Relief and Development Foundation (SRD) |
| --- | --- | --- | --- | --- |
| Year of establishment | **1998** | **1975** | **2012** | **2011** |
| Historical role prior to the start of the Syrian conflict | **A Syrian American Medical association operates as an international NGO. It implements medical education projects, medical campaigns, and medical conferences.** | **Relief and international development** | **Mental health, empowerment, capacity building and research projects** | **N/A** |
| Current role | **Supports an undergraduate midwifery and nursing health technical institute in Al-Atareb.**  **Supports the Syrian Board of Medical Specialities.**  **Implements many training initiatives.** | **Supporting the Academy of Health Sciences in Qah.**  **Supporting the medical residency program for twenty resident physicians at SBOMS through partnership with SEMA organization.**  **Offering 20 MSc. scholarships at Ankara Yildirim Bayezid University in Turkey in public health and health policies.**  **Training health directorates and organizations’ staff in epidemiology.** | **Mental health, empowerment, capacity building and research projects** | **Building capacity for the foundation staff in different health topics.**  **Inter-agency training.**  **Support a midwifery school.** |
| Area of expertise | **General medicine, specialist medicine, general nursing, specialist nursing, midwifery, special education for psychotherapists, special education clinical Psychologists** | **Medical relief and development** | **Mental Health and psychosocial support‏ (MHPSS)** | **Health, medical and protection projects. Including primary and secondary health care, specialized services, capacity building, supporting health system including networking, referral, non-communicable diseases (NCD), Infection prevention and control (IPC)** |
| Current interventions | **Professional diploma program in nursing and midwifery.**  **Intensive care physicians training,**  **Vocational training for psychological support workers and psychotherapists,**  **Health sciences students’ scholarship program in neighbouring countries.** | **Operational support for the Academy of Health Sciences and Medical Residency Program** | **Building managerial and leadership capacities and life skills**  **Protect and empower women,**  **children and youth.**  **High-quality capacity-building support in all required areas that support the realization of its vision: in particular, the program of advanced problem-solving, as per the third level of the pyramid of psychosocial interventions** | **WHO health programs including; SRH, NCD, IPC, Referral, mhGAP, CMR, IYCF, CMAN, community healthcare workers,**  **Neonatal and Childhood Illnesses trainings.**  **Supporting a midwifery school** |
| Aims of the current interventions | **Developing the health system in north-western Syria.**  **Providing the medical community with qualified medical specialist cadres,**  **Confronting all medical humanitarian crises with professionalism, experience, and international reliability** | **Supporting the health system and building blocks for early recovery phase.** | **Building the capacities of the non-specialized personnel, so that they can provide the same support for patients with moderate psychological complaints, in order to compensate for the large shortage of psychiatrists and psychotherapists.** | **Build and enhance the capacity for health provider to provide health services with high quality.** |
| Main challenges | **The lack of international Accreditation of Certifications,**  **Absence of governmental oversight bodies,**  **Decreased funding for medical educational institutions,**  **Unstable security situation** | **Security situation**  **Covid-19** | **Lack of support.**  **Doners’ tendency to give grants to large organizations that are not specialized in the field of mental health rather than to specialized organizations** | **Accessibility**  **Availability of sustainable funds and support**  **conflicts.** |
| Coping strategies | **local advocacy,**  **international advocacy,**  **Capacity building of civilian local medical authorities** | **-** | **-** | **-** |
| Recommendations | **Empowering the local medical community,**  **health system strengthening,**  **Supporting health facilities and supporting their independence** | **-** | **-** | **-** |
| Types of support needed | **Oversight of medical certificates,**  **Coordination between working humanitarian agencies,**  **Facilitating the work of organizations and the movement of employees,**  **Supporting health personnel in terms of security risks management.** | **-** | **-** | **-** |

**Table B: Detailed analysis of the NGOs supporting the MEHPT sector in northwest Syria (cont.)**

| NGO | David Nott Foundation | Union of Medical Care and Relief Organisations (Uossm) | Hand-in-Hand for Aid and Development (HIHFAD) | Independent Doctors Association (IDA) | The Syrian British Medical Society (SBMS) |
| --- | --- | --- | --- | --- | --- |
| Year of establishment | 2015 | 2012 | 2011 | 2015 | 2007 |
| Historical role prior to the start of the Syrian conflict | N/A | N/A | N/A | N/A | Supporting the Syrian doctors in the UK and in Syria.  Supporting ATLS courses. |
| Current role | surgical and trauma training for surgeons in the area | Training the institutions' cadres on the protocols recommended by the World Health Organization | Supports a network of Health facilities including 3 general hospitals, 2 maternity and children hospitals, 2 primary health centres, 3 Physiotherapy Centres, 1 TB Centre (the only one in Idleb ), and one prothesis Centre.  Through those health facilities HIHFAD provides practical training and residency courses in coordination with SBOMS.  In 2017, supported a project to qualify 120 student nurse assistants. | Supporting a training centre in BAS - Border side | Expertise advice through 200 members of consultants- Advanced surgical and trauma courses with coordination with the Royal College. |
| Area of expertise | surgery and surgical trauma training | Supports the establishment and operation of primary care centres at all levels, in addition to secondary and specialized care centres | Medical staff including General and specialized Doctors - Nurses - Midwives - Medical Technicians - Pharmacists - Lab Techs and other HCWs. | Healthcare | Advanced surgical and trauma training. |
| Current interventions | surgical courses with post graduate education for surgeons, educational online group, medical advice and surgical expertise. | Training the staff on the programs and protocols recommended by the organization  Global health, such as: IMCI, IYCF, NCD, CD, CMAM, MAM, PNA, ANC, FP, PFA, mfGAP, ...etc | Supporting a residency program in coordination with SBOMS | Supporting the provision of Primary, secondary and tertiary health care services in addition to blood bank and protection services integrated in the health centres |  |
| Aims of the current interventions | To train surgeons how to deal with trauma and surgical cases and provide up-to-date expert training and advice in this field. | Capacity building and improving the skills of the HCWs to be able to provide the required services to the beneficiaries on the highest quality | Filling the gap in qualified staff especially doctors  through supporting the residency programs. | Vocational and academic training to medical and paramedical staff |  |
| Main challenges | financial concerns, feasibility and security issues facing the foundation committee to reach these surgeons, no online platforms and Internet to be able to conduct this work. | Covid 19  Securing financial support from donors | Sustainability - The shortage of funds -Shortage of the qualified staff- unstable security conditions in the area | Scarcity of expert staff and shrinkage in the fund | Lack of financial independence- financial logistical complexities  . |
| Coping strategies | - | Networking with donors- securing financial requirements to support capacity building for each project | Advocacy for health Services - Advocacy for long term education programs to face the lack of human resources - Clear central SoPs and protocols to standardize the services and to create a unified model according to the needs rather than to the donors’ requests and attitudes | Providing academic and vocational training to  selected groups of auxiliary health workers to fill the gap in medical and paramedical staff | Working towards achieving financial independence and independent decision making as an association. |
| Recommendations | - | Prioritizing cadres’ capacity building | Coordination with other partners and official bodies such as the health cluster - supporting SBOMS in centralizing the education projects - | Diversifying the sources of  fund and establishing concrete medical training programs to fill in the shortage in medical staff | Improving communication with the local doctors- Carrying out in-depth needs assessments in the area- Coordination with local authorities |
| Types of support needed | - | Securing sustainable financial support- working towards accreditation | sustainable funds – Improving the quality of health services  - developing  post recovery projects to achieve sustainability | Supporting the medical education programs in the area. | Detailed needs assessments through a reliable health authority- Designing sustainable programs. |

**Table C: Detailed analysis of the NGOs supporting the MEHPT sector in northwest Syria (cont.).**

| NGO | Syrian Expatriates Medical Association (SEMA) | MIDMAR |
| --- | --- | --- |
| Year of establishment | 2012 | 2017 |
| Historical role prior to the start of the Syrian conflict | N/A | N/A |
| Current role | High quality medical education and training activities in all of the Syrian accessible areas including the besieged ones. Currently activities take place in North of Syria (including Olive Branch, Euphrates Shield, Peace Spring, Idlib and its countryside) and in Turkey (Hatay – Gaziantep).  Have supported 400 students to graduate, with remarkable advantage to have a suitable job opportunity after graduation.  SEMA is a member of:  - Health Cluster – Turkey Hub (WHO)  - Education Cluster – Turkey Hub  - Southeast Turkey Education Work Group  - Early Recovery and Livelihoods cluster  - Higher Education Working Group - Ankara | Previous experience within UOSSM that extended from 2013 to mid-2017, Providing a range of specialized services in CME including training programs, quality diplomas and response projects.  Implementation of service contracts of medical software for medical institutions operating in north-western Syria.  Currently, supporting a project to qualify 125 specialized nurses (intensive care - incubator care - surgeon assistant – dialysis)^[[1]](#footnote-1)^ |
| Area of expertise | Medical (all specialties) - Nursing - Paramedics - Midwifery - Laboratories - Physiotherapy - Orthotic and Prosthetics - Pharmacy technicians | CME- service contracts of medical software for medical institutions- specialised nurses’ education. |
| Current interventions | A- Continuing Medical Education (CME)^[[2]](#footnote-2)^.    B- Vocational Health Education:  1- Supporting the Academy of Health Sciences: Specialized programs (nursing, physiotherapy, and emergency medicine for 127 students) in 24 months education programs, our programs are accredited by ACQUIN (Accreditation Certification and Quality Assurance Institute – Germany)   2- SEMA Vocational Health Education Centre: blended learning programs (nursing and midwifery for 100 students) in Afrin, which are accredited by the Turkish Ministries of National Education and Health.   3- Master programs of Health Policy and Global Health for 20 Syrian health care providers working within the Syrian humanitarian health context (Syria and SE Turkey). These scholarships are provided in collaboration with Idleb Health Directorate, Ankara Yildirim Beyazit University, and the Qatari Red Crescent.  4- SEMA conducted two strategic cooperation agreements to support and develop Gaziantep University’s Health Institutions (four majors) in Jarablus inside Syria, and to collaborate through a comprehensive continuing medical education program for healthcare staff delivering services in all specialties in North of Syria  5- Safe Neonatal Emergency Transport Program (SENT Program) in collaboration with Idleb Health Directorate and the World Health Organization   6- The Paediatric residency lecture Series: Seeks to help 75 Syrian (paediatricians/ paediatric residents) solving the problem that neonatal/newborn healthcare services inside Syria lack of qualified specialists in the hospitals of the safe zone northern of Syria (Aleppo and Idleb countryside). The program is composed of a prolonged lecture series, practical supervision, and an internship period (planned) within Gaziantep University Hospital. The lectures cover different Paediatric subspecialties with approximately a lecture/week, 62 lectures were provided within the last 11 months.  7- Currently SEMA conducted a strategic cooperation agreement with the Turkish Red Crescent to establish a new vocational health education institution to qualify specialized health care personnel especially the psychosocial services providers) in Kafarlosin – harem district - Idleb inside Syria   8- Medical Education Vocational Diploma Program: for lecturers, trainers, instructors, and supervisors to build their capacities and raise the quality of the health education services they provide. This initiative is in cooperation with Aleppo University in the Liberated Areas   C- Health research and medical translation | supporting a project to qualify 125 specialized nurses (intensive care - incubator care - surgeon assistant – dialysis)^[[3]](#footnote-3)^ |
| Aims of the current interventions | 1- Build the health care providers' capacity with life-saving training. 2- Raising awareness about proper medical procedures.  3- Improve the quality of emergency care and rehabilitation services.  4- Developing health vocational institutions and graduating qualified health care providers.  5- Secure sustainability and youth empowerment through academic accreditation and meeting the needs of labor market.  6- Encourage the scientific research culture.  7- Support the health care system recovery. |  |
| Main challenges | Multiple working authorities and stakeholders   Health education initiatives’ funds are difficult to secure  Multiple and different accreditation and endorsement  Low health education quality in general and the need to requalify a lot of other stakeholders' graduates  Lack of organization and governance within this sector  Lack of experience of the lecturers, trainers, instructors, and supervisors | Lack of stable financial support- geopolitical challenges- Lack of standardization of training assessment and certification. |
| Coping strategies | - | - |
| Recommendations | - | - |
| Types of support needed | - | - |

**Table D: Detailed undergraduate MEHPT university analysis^[[4]](#endnote-1)^**

|  | Free Aleppo University(30) | Nahda Private University(31) | Al-Hayat Private University |
| --- | --- | --- | --- |
| Type of health science institution/school: | Undergraduate  faculty of medicine, faculty of dentistry, faculty of pharmacy, faculty of health sciences, anaesthesia, nursing, and midwifery institutes. | Undergraduate  faculty of medicine, faculty of dentistry, faculty of pharmacy, faculty of health sciences.  Pharmacy, radiology, orthodontics, paediatric nursing, biochemistry, and midwifery institutes | Undergraduate  Faculty of Nursing, Faculty of midwifery, Aesthesia and physiotherapy institutes |
| Supporting authority- Accreditation | Council of Higher Education in the liberated areas / Syrian Interim Government. | Council of Higher Education in the liberated areas / Syrian Interim Government. | - |
| Geographical location | Mari’ -north Aleppo- Syria | Izaz-Ephorate Shield- Aleppo- Syria | Acrabat- Idlib- Syria |
| Funds available | No information available | 300 $ per student annually- 300, 000 $ annually in total | No information available |
| Funding sources | Tuition fees paid by the students | Donations + Tuition fees paid by the students | Medical Education Board |
| Funds unavailable | 30% of the required funds is unavailable | No information available | No information available |
| Registration fees/scholarship for students per year | 250$ per student annually- some students can get scholarships | 50$- 3000$ per year (student fees post the scholarship) | Free |
| Number of deanery staff of institution/school | 2 | 6 | 10 |
| Qualifications of deanery staff in each medical school: | MSc- PhD | MSc- PhD | MSc- PhD- Bachelors-Diploma |
| Training available for deanery staff | Integrating technology into medical education, quality control, assessment, decision making and problem-solving skills, strategic planning, university management, time management, disaster management | Online teaching- Teaching strategies | Microsoft Office- TOT |
| Training uاunavailable for deanery staff | Medical education methods, assessment, scientific research, higher education development | Curriculum planning- Medical education strategies- up-to-date clinical technologies- health management | Management- HR |
| Managerial staff available | 8 | 14 | 15 |
| Managerial staff unavailable | 2 Undergraduate staff, 2 exams centre, 8 heads of departments | 10 | Physiotherapy |
| Training available for managerial staff | Quality improvement, management, correspondence training | Distance education / student admission – Exams management /  / | Microsoft Office- TOT |
| Training unavailable for managerial staff | Management training, quality improvement | Mental wellbeing support for students | HR |
| Number of terms at institution/school | 2 terms/year | 3 terms/year | 2 terms/year |
| Number of academic years till graduation | 6 years | 4 years- health institutions  8 years- health faculties | 4 years |
| Number of students | More than 2100 students, including faculty of medicine, faculty of dentistry, faculty of pharmacy, Anaesthesia, nursing, and midwifery institutes. | 1000 students | Faculty of Nursing 242 , Faculty of midwifery 111 , Aesthesia institutes 86 , Physiotherapy institute199 |
| Modules taught till graduation | 72 | 26 modules- health institutes  62 modules- health faculties | 45- 48 modules |
| Modules not taught in each year | Rheumatology, neurophysiology, endocrino-physiology | Histology- pathology- physiology- biology- genecology | none |
| Reason of untaught modules | Unavailable teaching staff | Unavailable teaching staff- unavailable suitable lab | N/A |
| Number of teaching staff – in person and diaspora | 30 in person, 10 diaspora | 50 | 41 in person 7 diaspora |
| Qualification of teaching staff for each module | Bachelors- MSc- PhD | Bachelors- MSc- PhD | Bachelors- MSc- PhD- Diploma |
| Training available for teaching staff | Medical education planning, improving education | Distance education | Teaching strategies- assessment- scientific research- communication skills |
| Training unavailable for teaching staff | Assessment | Medical Education strategies | office HR |
| Teaching approach | Seminars, lab sessions, lectures, interactive sessions, clinical training | Seminars, lab sessions, lectures, interactive sessions, clinical training | Seminars, lab sessions, lectures, interactive sessions, clinical training |
| Curriculum implemented | Damascus university curriculum | Blended- local and international curriculum | Blended- Tishreen University curriculum and local curriculum |
| Infrastructure building and equipment available to aid teaching | Lecturing theatres, seminar rooms, labs, hospital training | Labs- contracts with hospitals- online platform | projectors- screens- whiteboards-mannikins |
| Infrastructure and building and equipment unavailable to aid teaching | Library, labs | University hospital- quality labs- biochemistry lab | none |
| Research module available? Which year? Unavailable? Reason? | Two compulsory modules in the third and fifth years for the faculties students | Optional module | Two compulsory modules-fourth year |
| Work opportunities post-graduation | One cohort graduated so far, almost 100% had jobs. | Almost 100 % - however facing difficulties to gain governmental accreditation | 80% nursing, 65% anaesthesia, 45% physiotherapy, 70 % midwifery |
| Enrolment criteria | High school score, and an enrolment exam | High school score | High school score, and an enrolment exam and physical fitness to practice |
| Sustainability of educational programme | Ongoing since 2015 | Only in 2017 in-person teaching was paused due to Covid19 and was held online | Ongoing since 2015-2016 |
| Assessment criteria | Theory and practical exams | Several criteria | Theory and practical exams |
| Clinical training available | yes | yes | yes |

**Table E: Detailed undergraduate MEHPT university analysis (cont’d)^[[5]](#endnote-2)^**

|  | Health Sciences Academy(32) | Mary Private University(33) | Idlib University(35) | Al-Shamal Private University^[[6]](#footnote-4)^(34) |
| --- | --- | --- | --- | --- |
| Type of institution school: | Undergraduate  Paramedics  nursing, and  physiotherapy institutes | Undergraduate  Faculty of dentistry, orthodontics, and dentists’ assistants’ institutes | Undergraduate  faculty of medicine, faculty of dentistry, faculty of pharmacy, nursing, midwifery, emergency, and anaesthesia health institutes | Undergraduate  Faculty of Medicine, faculty of pharmacy, orthodontics institute, pharmacy institute, and anaesthesiology institute |
| Supporting authority- accreditation | - | - | Ministry of Higher Education in the Salvation Government. | - |
| Geographical location | Quah- Idlib- Syria | Hazano- Idlib-Syria | Idlib-Syria | Sarmada-Idlib-Syria |
| Funds available | No information available | No financial support available | No information available | No information available |
| Funding sources | SEMA | Tuition fees paid by the students | Tuition fees paid by the students | Tuition fees paid by the students |
| Funds unavailable | none | none | No information available | No information available |
| Registration fees/scholarship for students per year | Free | 1200$-1500$  Waivers available for war victims and teaching staff sons/daughters, and siblings’ discounts | 200$-250$ per student annually | 800$-1400$ per student annually |
| Number of deanery staff of institution/school | 5 | 5 | No information available | No information available |
| Qualifications of deanery staff in each medical school: | MSc | MSc- PhD | No information available | No information available |
| Training available for deanery staff | Teaching methods, infection control, quality control | None | No information available | No information available |
| Training uاunavailable for deanery staff | none | Teaching strategies and methods | No information available | No information available |
| Managerial staff available | 17 | 11 | No information available | No information available |
| Managerial staff unavailable | none | none | No information available | No information available |
| Training available for managerial staff | Time management- quality control- ACDL | none | No information available | No information available |
| Training unavailable for managerial staff | none | Management courses- archiving and electronic archiving training. | No information available | No information available |
| Number of terms at institution/school | 2 terms/year | 2 terms/year | No information available | 2 terms/year |
| Number of academic years till graduation | 2 years | 5 years + Internship year | No information available | No information available |
| Number of students | Paramedics 40, Nursing 42 , Physiotherapy 45 | 500 in total | More than 2000 students, including faculty of medicine, faculty of dentistry, faculty of pharmacy, nursing, midwifery, emergency and anaesthesia health institutes. | Faculty of Medicine, faculty of pharmacy, orthodontics institute, pharmacy institute, and anaesthesiology institute  (no information available on the number of students) |
| Modules taught till graduation | 28-30 modules | 29 modules- health institutes  69 modules- health faculty | No information available | No information available |
| Modules not taught in each year | none | none | No information available | No information available |
| Reason of untaught modules | N/A | N/A | No information available | No information available |
| Number of teaching staff - in person and diaspora | In person22 | 34 in-person | No information available | No information available |
| Qualification of teaching staff for each module | Bachelors- MSc | MSc- PhD- Diploma | MSc- PhD | No information available |
| Training available for teaching staff | Teaching strategies- scientific research- English language- Turkish language, | none | No information available | No information available |
| Training unavailable for teaching staff | Clinical research | Teaching methods and strategies- Scientific research methodology | No information available | No information available |
| Teaching approach | Seminars, lab sessions, lectures, interactive sessions, clinical training, and simulation. | Lectures and traditional teaching approaches | Seminars, lab sessions, lectures, interactive sessions, clinical training, conferences | Seminars, lab sessions, lectures. |
| Curriculum implemented | Blended- Tishreen University curriculum and local curriculum | University of Aleppo curriculum | based on translations from textbooks, and Syrian universities. | No information available |
| Infrastructure building and equipment available to aid teaching | Lecture theatres equipped with projectors- screens- whiteboards-mannikins | Lecture theatres and labs | Lecturing theatres, seminar rooms, labs, university hospital training | No information available |
| Infrastructure and building and equipment unavailable to aid teaching | none | none | No information available | No information available |
| Research module available? Which year? Unavailable? Reason? | No- intense curriculum | No- lack of teaching staff and expertise to design curriculum | Yes- Research modules in medical school, dentistry and pharmacy | No information available |
| Work opportunities post-graduation | 75 % | Limited | 97% enrolment | No information available |
| Enrolment criteria | High school score, and an enrolment exam and physical fitness to practice | High school score | High school score | High school score |
| Sustainability of educational programme | Ongoing since 2015 | Ongoing since 2016 | Ongoing since 2015-2016 | Ongoing since 2015 (was under the name Oxford University – Syria) |
| Assessment criteria | Theory and practical exams | Theory and practical exams | Theory and practical exams | Theory and practical exams |
| Clinical training available | yes | yes | yes | No information available |

1. ) https://www.uossm.org/training https://midmar.org/developing-activities-training/ [↑](#footnote-ref-1)
2. 1- https://www.youtube.com/c/SEMAEducation

   2- https://riada-education.com/?&lang=en [↑](#footnote-ref-2)
3. ) https://www.uossm.org/training https://midmar.org/developing-activities-training/ [↑](#footnote-ref-3)
4. “no information available” fields indicate where participants refused to fill or were unsure about information. [↑](#endnote-ref-1)
5. “no information available” fields indicate where participants refused to fill or were unsure about information. [↑](#endnote-ref-2)
6. Information acquired from the university official website [↑](#footnote-ref-4)
